# The visual perception of long outdoor distances

**DOI:** 10.1038/s41598-024-53835-1

**Published:** 2024-02-08

**Authors:** J. Farley Norman, Jessica L. Lewis, Alejandro B. Ramirez, Emily N. Bryant, Payton Adcock, Roseanna D. Peterson

**Affiliations:** 1 Department of Psychological Sciences, Ogden College of Science and Engineering, Western Kentucky University, 1906 College Heights Blvd. #22030, Bowling Green, Kentucky 42101-2030 USA; 2 Carol Martin Gatton Academy of Mathematics and Science, Bowling Green, Kentucky USA

**Keywords:** Perception, Human behaviour

## Abstract

Many previous studies have investigated visual distance perception, especially for small to moderate distances. Few experiments, however, have evaluated the perception of large distances (e.g., 100 m or more). The studies that have been conducted have found conflicting results (diametrically opposite conclusions). In the current experiment, the functions relating actual and perceived distance were obtained for sixteen adult observers using the method of equal appearing intervals. These functions relating perceived and actual distance were obtained for outdoor viewing in a typical University environment—the experiment was conducted along a sidewalk adjacent to a typical street where campus buildings, trees, street signs, etc., were visible. The overall results indicated perceptual compression of distances in depth so that the stimulus distance intervals appeared significantly shorter than the actual (physical) distance intervals. It is important to note, however, that there were sizeable individual differences—the judgments of half of the observers were relatively accurate, whereas the judgments of the remaining half were inaccurate to varying degrees. The results of the experiment demonstrate that there is no single function that describes how human observers visually perceive large distance intervals in outdoor environments.

## Introduction

When we look out at the world, we see buildings, trees, people, and a great variety of environmental objects, each separated from others by particular distances. The resulting spatial relationships seem clear to us, but when we make judgments about apparent distance magnitudes between objects (or between ourselves and an object) the results quickly become complicated. One interesting fact is that distance judgments are context dependent. For example, distances in indoor environments are perceived differently than analogous distances outdoors^1–3^. Nevertheless, many studies in both indoor and outdoor environments have found that at short^4–7^ and medium^8–15^ distances, distance intervals oriented in depth are frequently compressed and appear significantly shorter than they exist in reality. Such studies indicate that space is subjected to an *affine* transformation^4,7,14^ during the process of perception. Gilinsky^12^ has succinctly described this view by pointing out (p. 461) that “Visual space and physical space are not identical… One is a distorted transformation of the other”. A number of studies have found that the distortions of space that occur during perception are not affine. Instead, perceived space is curved in either an *elliptic* or *hyperbolic* fashion^4,8,16–18^. Other findings less commonly obtained are that visual space is either nonmetric^19^ in nature or approximately Euclidean^20^.

Few existing studies have evaluated the perception of very long distances beyond 100 m. Those that have been conducted produced highly conflicting outcomes. Both Kuroda^21^ and Da Silva^22^ found the frequently observed perceptual compression of distances in depth (i.e., those aligned along an observer’s line of sight). As an example, consider Kuroda’s Experiment 2—this experiment used the method of equal appearing intervals and took place along a 127 m stretch of road outdoors. A reference rod (1 m long) was placed on the ground 7 m from each observer. The observers’ task was to indicate where an experimenter should place a second rod further in depth so that the distance between it and the reference rod appeared identical to the initial distance (the 7 m between the observer and reference rod). This process continued three more times, creating third, fourth, and fifth distance intervals that all appeared equal in magnitude to the initial two. Perceptually, all of the five distance intervals appeared equal, but Kuroda found that each constructed distance interval became physically larger the farther away from the observer it was located (see p. 214 of Kuroda^21^). Such results indicates that physical space was being compressed more and more during the process of perception as the judged distances in depth were located farther and farther away (e.g., if a physical interval of 14 m located farther away looks equivalent to a 7 m interval closer to an observer, strong perceptual “compression” of the farther interval is occurring).

In contrast to the perceptual compression of distances in depth found by Kuroda^21^ and Da Silva^22^, Purdy and Gibson^23^ found relatively accurate visual perception of distance interval magnitudes. In their outdoor experiment, observers bisected (or trisected) long distance intervals of 137 to 274 m and found that the resulting errors in bisection (or trisection) were only 3.1 percent of the total distance extent on average. Purdy and Gibson therefore concluded (p. 380) by saying “Observers can divide stretches of distance (up to 300 yd.) into halves or thirds with very good accuracy. Perceived magnitudes of distance appear to correspond well with physical magnitudes of distance”.

The purpose of the current experiment was straightforward. Given the diametrically opposite findings of Purdy and Gibson^23^ and Kuroda/Da Silva^21,22^ (that very long distances in depth are completely accurate *or* highly distorted), we sought to determine which of these previous research findings is more correct. The experimental task used by Purdy and Gibson (bisection and trisection) was a simplification of Kuroda’s task, which could be called quintsection (observer creates five equal-appearing distance intervals). The advantage of Kuroda’s method is that given the data, one can derive the entire function relating perceived and actual distance (e.g., see Kuroda’s Fig. 10). In the current study, we adopted the more complete method of Kuroda (and even extended it so that our observers had to equate six distance intervals in depth).

## Method

### Apparatus

The adjustable cones used as distance markers were 44 cm tall and 27 cm in diameter at their base. The cones were highly visible (bright orange) and were the same as those used in a previous study^13^. Communication between researchers during the experiment over the 100 to 300 m distance was by UHF (ultra-high frequency, 462.575 MHz) radio (Midland X-Talker radios, model T31VP). The resulting experimental data were analyzed using SPSS (IBM Corp.) on an Apple iMac computer.

### Experimental stimuli

**Figure 1:**
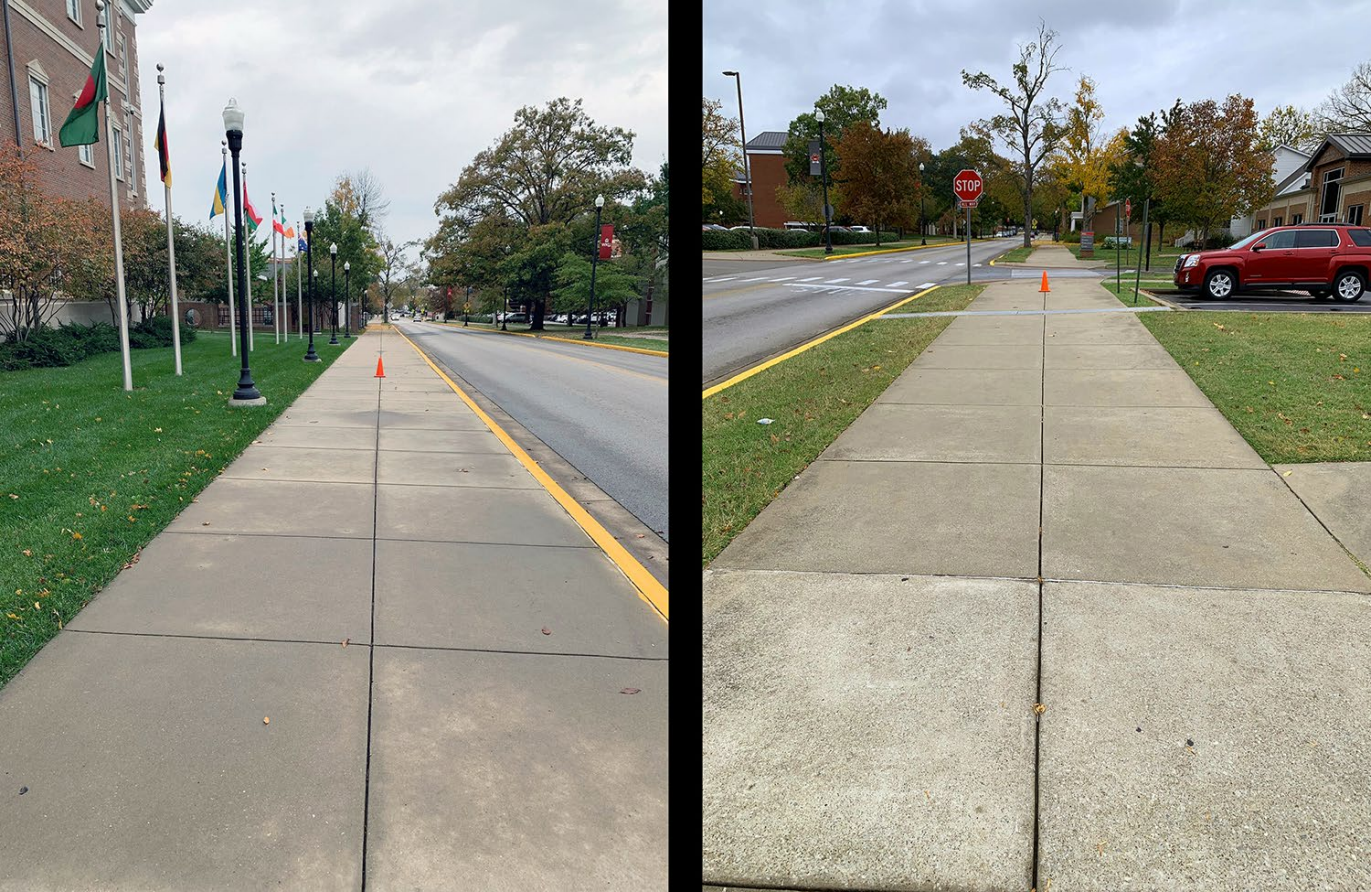
Photographs of the sidewalk used in the experiment, taken from the observers’ vantage point. Observers either stood at the top of the slight incline and looked down (left panel) or they stood at the bottom of the incline and looked up (right panel). These photographs were taken by the first author (J.F.N.) using an Apple iPhone XR digital camera.

The stimulus distance intervals were created along a sidewalk on the east side of Normal Drive, located on the campus of Western Kentucky University. The approximately 300 m section of sidewalk that was used for the experiment was straight (i.e., uncurved, see Fig. 1). There was a slight incline of 0.15 degrees, so that the observers looked at distance extents that were slightly inclined either downwards or upwards. The experiment was always conducted in the daytime so that the viewing conditions were full cue. It is readily apparent from Fig. 1 that the horizontal divisions between adjacent sections of the sidewalk were visible up to the reference cone located at 17 m. However, it is very important to note that they were not visible at further distances past the reference cone, and thus could not affect performance on the experimental task. When the observers were looking at the stimulus distance intervals down the sidewalk they could see neighboring campus buildings, trees, etc. It is obvious from an inspection of Fig. 1 that the sidewalk and neighboring street (Normal Drive) produced large amounts of linear perspective.

### Procedure

As in the study by Kuroda^21^, we used the method of equal appearing intervals. A random half of the observers stood at the top of the slight incline and looked down the sidewalk, while the remaining half of the observers stood at the bottom of the incline and looked up (Fig. 1). A reference cone (visible in Fig. 1) was first placed at a distance of 17 m from the observer (it is important to note that the observers did not know that this initial distance extent was 17 m; the observers were also not allowed to see the placement of the reference cone). The observers then told an experimenter where to place a second cone so that the distance from the second cone to the reference cone appeared identical in magnitude to the egocentric distance from the observers to the reference cone. The observers then directed an experimenter where to place a third cone so that the apparent distance from the third cone to the second equaled both the initial distance to the reference cone and the distance separating the reference and second cones. This process was repeated an additional three times. At the end of this procedure, the observers had created a total of six distance intervals in depth (i.e., along their line of sight) that all appeared equal in magnitude from their point of view. From arrival at the laboratory until data collection was complete, the total time required for each observer took about 45 min. No feedback about performance was ever provided to an observer until their judgments were complete.

### Observers

In the current investigation, we obtained data from a total of 16 observers, who were primarily faculty or students at Western Kentucky University. All except one (the first author, JFN) were completely naïve and had no knowledge of the specific goals of the experiment. The observers had good visual acuity: the acuity of the observers (measured with a PrecisionVision 2195 ETDRS eye chart) was − 0.11 LogMAR (log minimum angle of resolution; zero LogMAR represents normal visual acuity, while negative and positive values represent better than and worse than normal acuity, respectively). The study was approved by the Institutional Review Board of Western Kentucky University, and each participant signed an informed consent document prior to testing. Our research was carried out in accordance with the Code of Ethics of the World Medical Association (Declaration of Helsinki).

## Results

**Figure 2:**
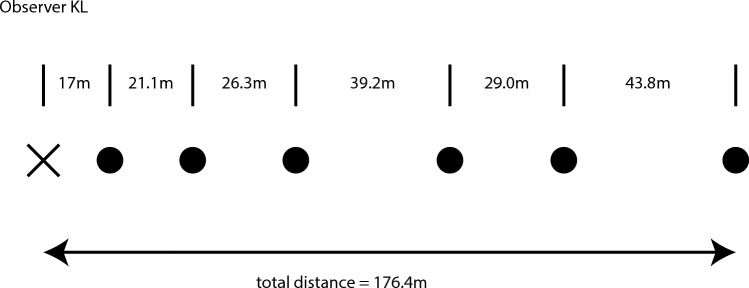
A depiction of the individual results of Observer KL. The observer’s vantage point is marked with an X, while the filled circles indicate the final adjusted positions of the orange distance markers. From the observer’s point of view, all six of these distance intervals appeared equivalent in magnitude.

**Figure 3:**
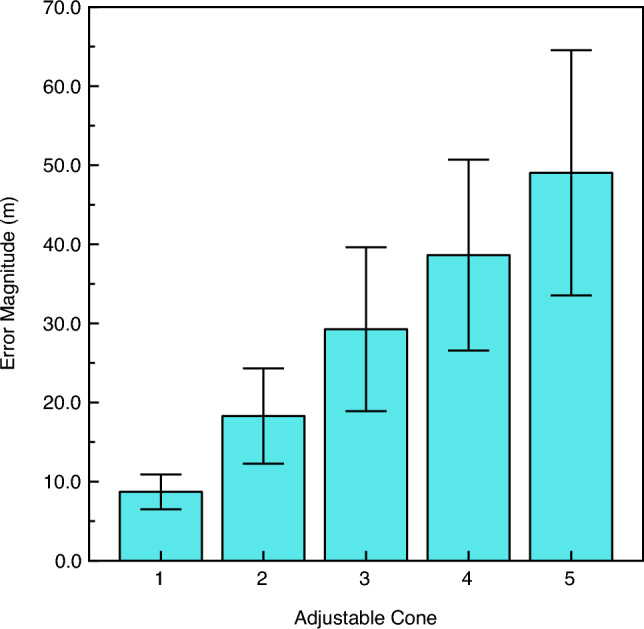
The observers' overall error magnitudes in placement of the distance markers (i.e., orange cones) during the distance interval adjustment task. The first adjustable cone (i.e., Adjustable Cone 1) was the closest one to the observers, while the fifth adjustable cone (i.e., Adjustable Cone 5) was farthest from the observers. The average errors were all in the positive direction (i.e., placement of the cones farther than they would be located during accurate performance). The error bars indicate ± 1 SE.

**Figure 4:**
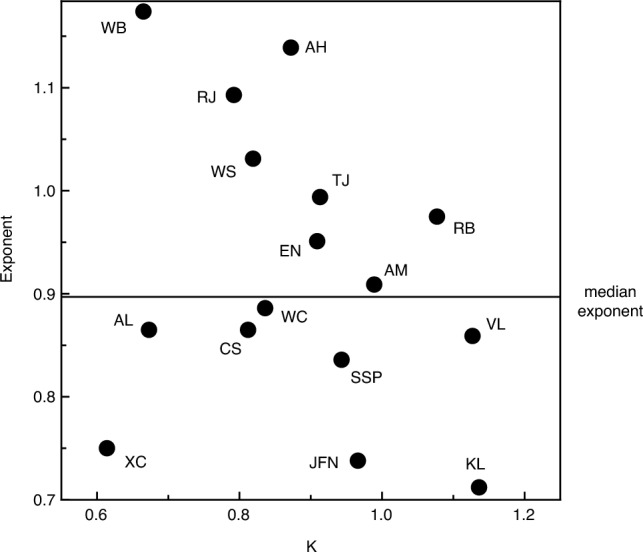
Each observer’s data was fit with the best-fitting power function, Dʹ = K * D^n^ (see text). This plot illustrates the best-fitting K and exponent (n) values for each of the individual observers.

**Figure 5:**
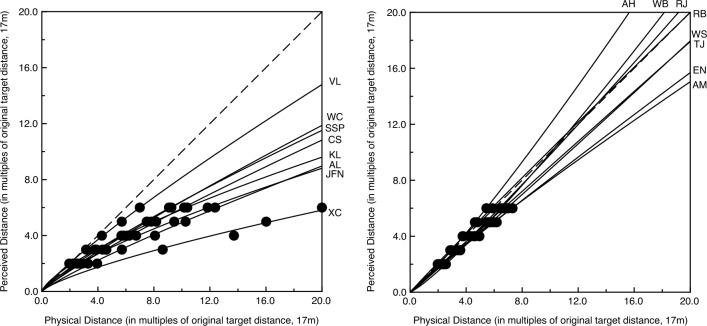
The individual observers’ functions relating perceived and physical distance (Dʹ = K * D^n^). The dashed line indicates accurate performance.

Various aspects of the results are shown in Figs. 2, 3, 4 and 5. A top-down view of a typical observer’s judgments (observer KL) is shown in Fig. 2. It is important to remember that once the distance markers (i.e., the orange cones) had been appropriately adjusted by the observers, all of the intercone distances appeared equivalent. It is readily apparent that for this observer there was a general increase in the magnitude of the intercone distances as the overall distance from the observer increased. The furthest intercone distance, for example, was physically 2.6 times larger than the initial 17 m distance to the reference cone, but those two distance intervals looked equivalent to observer KL. Such judgments were typical for many of the other observers. Figure 3 plots the average error magnitudes for all observers (the errors are relative to where the cones should have been placed, if performance had been accurate; i.e., all intercone distances would be 17 m given accurate performance). In this context (Fig. 3), a negative error would mean that a cone was placed too near the observer; a positive error indicates that a cone was placed too far. It is important to note from Fig. 3 that overall, the errors were positive and increased in magnitude the farther the distance from the observers. This effect of increasing (positive) error magnitude as a function of overall distance was significant (F(1.043, 14.6) = 8.7, p = 0.01; η^2^_p_ = 0.38) according to a 2 × 5 split-plot analysis of variance (ANOVA). We used a conservative test, adjusting the degrees of freedom according to the methods of Greenhouse and Geisser^24^. Since the overall errors (Fig. 3) are positive and increase in magnitude with distance, the overall results of our experiment demonstrate increasing perceptual compression of distance intervals in depth at farther and farther distances (e.g., see Fig. 2), as described by Gilinsky^12^. There was no effect (F(1, 14) = 1.4, p = 0.26; η^2^_p_ = 0.09) of the direction in which the observer faced (i.e., looking down the slight incline or looking up the slight incline; see Fig. 1). The interaction between viewing direction and distance was not significant either (F(1.043, 14.6) = 0.7, p = 0.42; η^2^_p_ = 0.05).

Previous research^21,22,25,26^ has demonstrated that visually perceived distance can be well described by a power law (1), where Dʹ refers to perceived distance and D to physical distance.

1 $${\text{D}}^{\prime } = {\text{K*D}}^{{\text{n}}}$$

Like Da Silva^22^, we found the best-fitting power function for each individual observer. The resulting K and exponent (n) values are shown in Fig. 4. It is readily apparent that each individual observer’s function was described by a different pair of parameter values. Exponents of 1, < 1, and > 1 would indicate linear, logarithmic, and exponential relationships (between perceived and physical distance), respectively. The median exponent value for our 16 observers was 0.897. The functions relating perceived and actual distance are shown for each individual observer in Fig. 5; the left panel plots the observers’ functions with exponent values less than the median, while the right panel plots the analogous functions with exponent values greater than the median. A one-sample t-test conducted upon the exponent values indicates that the observers’ exponents deviate significantly from 1.0 (t(15) = 2.21, p = 0.04, 2-tailed), once again demonstrating that overall, our observers’ functions demonstrate perceptual compression of distances in depth. It is very important to note, however, that our observers differed widely in the degree of perceptual compression. Indeed, some of our observers’ judgments indicated that their relationship between perceived and physical distance was essentially linear (see right panel of Fig. 5).

## Discussion

In 1985, Da Silva^22^ investigated the perception of long distances—for example, in his Experiment 1, observers verbally estimated distances to targets within a 30 × 300 m field. After finding the best-fitting power function for each individual observer, he found that the average exponent (n) value was 0.84, indicating perceptual compression of distances in depth (with the compression increasing in magnitude at farther and farther distances). In 1971, Kuroda’s^21^ Experiment 2 used the method of equal appearing intervals to investigate the perception of distance intervals along a 127 m span of road. Kuroda’s results agree with those of Da Silva (see Fig. 10 of Kuroda^21^). In contrast to these studies, Purdy and Gibson’s^23^ findings were completely contradictory. Their experiment utilized bisection and trisection of long distance intervals (e.g., up to 274 m) and they found accurate performance, with no systematic distortions of perceived distance at all (e.g., see Fig. 2 of Purdy and Gibson).

The results of our current experiment agree with both Kuroda and Da Silva^21,22^ on the one hand and Purdy and Gibson^23^ on the other. While our overall results (Fig. 3) are consistent with the perceptual compression of distances aligned in depth and are thus similar to the results of Kuroda and Da Silva, we also found sizeable individual differences, such that many observers possessed essentially linear relationships between perceived and actual distance (in accordance with the results of Purdy and Gibson). Indeed, if we divide our observers by best-fitting exponent values (according to the median exponent, see Fig. 4), we find that half of our observers possess functions that agree with Kuroda and Da Silva (left panel of Fig. 5) with the remaining half of our observers exhibiting essentially linear relationships, reflecting relatively accurate performance (right panel of Fig. 5).

If the results of our current experiment are valid (that in the general population, significant individual differences exist, such that some people possess linear relationships between physical and perceived distance while for others, perceived distances in depth are compressed to greater and greater degrees as overall distance increases), then why did Kuroda^21^, for example, not find any evidence of accurate performance? Similarly, why did the many judgments in the Purdy and Gibson^23^ study not indicate any perceptual compressions of in-depth distance intervals? It is probably important to note in this context that only five observers participated in Kuroda’s experiments—with that sample size, it would be difficult to find much evidence of sizeable individual differences. With regards to Experiment 1 of Da Silva^22^, the sample size was excellent, but given the reporting of the results, it is difficult to evaluate the individual differences that did occur. Da Silva said that one group of observers had an average exponent value of 0.84 with a standard deviation ( ±) of 0.14. Assuming that the exponents for the 30 observers in this condition were normally distributed (and we do not know that for sure), then about 95 percent of these exponents would have magnitudes between 0.56 and 1.12. If true, this range of exponents (obtained by Da Silva) would actually be consistent with the range of exponents obtained in our current experiment (see Fig. 4). From the description of the method in their published article, it is impossible to understand some of the critical methodological details of the study by Purdy and Gibson (who used bisection and trisection tasks to evaluate distance perception). When describing their task (p. 375), these authors said “*The O was brought to his station point, given his instructions, and asked to turn his back while the markers were set up. When these were in place and the bicycle at the starting point of the stretch to be divided, E asked O to turn and judge when the moving target had reached the division point of the specified fraction*”. In this quote, O refers to the observer, while E refers to one of the experimenters. Unfortunately for us today, we do not know what actual instructions were provided to the observers for *how* to perform the bisection or trisection. Were the observers in the Purdy and Gibson study instructed to base their bisection or trisection judgments totally upon visually perceived distance per se and to not cognitively estimate or calculate the distance midpoints or thirds? There is simply no way to know. It is probably very important to note that all of the observers who participated in this 1955 experiment were military recruits and thus probably had or were undergoing basic marksmanship and/or range estimation training. They were thus probably trained to cognitively estimate distances for the purposes of accurately shooting battlefield targets or for dropping bombs at specific targets at particular distances, etc. In their article, Purdy and Gibson said (p. 375) that “*All O’s were Airmen in basic training at Sampson Air Force Base*”. If the observers in this experiment were using their military range training to cognitively estimate the distance intervals^27^ in order to make their judgments, this could explain why Purdy and Gibson only found accurate performance for their bisection and trisection tasks. We have used bisection tasks in our own previous research (involving judgments of shorter distances) and accurate performance is not typically obtained^1,2^; other researchers, such as Lappin, Shelton, and Rieser^3^ and Gilinsky^12^, have also documented inaccuracies in visual distance perception using a bisection task.

In order to properly evaluate and interpret the current results, there are additional complexities that must be explicitly considered. First, it is very important to note that different experimental tasks can produce contradictory outcomes even for single individual observers. For example, consider the results of Experiments 1 and 2 of Norman, Crabtree, et al.^8^. In Experiment 1, the observers adjusted the lengths (distance intervals) of two sides of a triangle outdoors until the triangle appeared equilateral (the one fixed side of the triangle was either 2 m or 15 m, so that the observers created either large or small equilateral triangles in depth). In Experiment 2, the observers performed a totally different distance-related task: they adjusted the magnitude of a distance interval oriented in depth until it appeared equivalent to a fronto-parallel distance interval. In one condition in Experiment 1 (binocular viewing of the large triangles), the judgments of observers MSH and JFN were consistent with Euclidean geometry, while in the match depth to width task of Experiment 2, the judgments of those same individual observers were consistent with a sizeable affine compression of distances in depth (so that a physical distance of about 6 m in depth appeared to be equivalent in extent to a 4 m fronto-parallel distance interval. The observers’ entire geometry of visual space changed as the task changed—in both experiments, observers adjusted the distance intervals defining a triangular configuration of poles (position markers) in the same grassy field outdoors. In addition, consider the judgments of observer JDG—in the match depth to width task in Experiment 2, his judgments were consistent with Euclidean geometry, but his performance for the large triangles in the equilateral triangle task consistently indicated that his geometry of visual space for that task was *hyperbolic*. Overall, in Experiment 1 (when the judgments of all observers were considered), the observers’ geometry of visual space depended to a large extent upon the size of the stimulus triangle. For the small triangles (2 m fixed side), the observers’ judgments were consistent with *elliptic* geometry (a non-Euclidean geometry analogous to the curvature of a hemisphere). In contrast, those same observers’ judgments became consistent with *hyperbolic* geometry (a non-Euclidean geometry analogous to the curvature of a horse saddle, convex in one direction and concave in a perpendicular direction). For more background on the curvature of visual space and similar results, see Koenderink, van Doorn, and Lappin^16^. These results indicate something really important: the quantitative *and* qualitative outcome of an experiment evaluating visual distance perception depends not only upon the individual observer and the physical extent of relevant stimulus distances, but also upon other factors that should be irrelevant if human observers could visually perceive distance magnitude per se (e.g., choice of task^8^, the overall size of the stimulus configuration^8,16^, large versus small, the orientation of the stimulus distances relative to the line of sight^6,9,11^, whether the environment is indoor or outdoor^1–3^, presence or absence of linear perspective^1,28^, whether a target object on outdoor terrain is viewed across a gap or across a change in surface texture^29^, etc.). Many factors other than physical distance per se influence our human judgments of environmental distance. In their 1967 article, when referring to the power law earlier mentioned (Eq. (1) in the results section), Baird and Biersdorf^6^ said (p. 164) “it would be unwise to accept a single exponent as representative of a general function for either size or distance judgments. The experimental method critically affects this value, *even when stimulus conditions are practically constant*”.

For the reasons just mentioned, it is difficult to make definite (or general) statements about the “perception” of distance per se. To do so requires converging operations. Despite differences in task, overall distance range (long, medium, short, very short, etc.), environmental setting, etc., there are some very important outcomes that are qualitatively the same across a wide variety of tasks, studies, contexts, and overall distances investigated. Consider the following. An outdoor study by Teghtsoonian and Teghtsoonian^30^ included one condition beyond 100 m. The researchers in this study utilized a technique quite different from ours—free-modulus magnitude estimation. Even though our method of equal appearing intervals was totally different, our findings were essentially identical. In the Teghtsoonian and Teghtsoonian experiment, the observers in that long-distance condition had an average exponent (see our Eq. (1) in the results section) of 0.85, reflecting overall perceptual compression of environmental distance. Wagner^14^ used a different type of magnitude estimation for medium distances (up to 40 m) and also found results consistent with perceptual compression. After obtaining the observers’ data and resulting computational modeling of the results, Wagner concluded by saying (p. 489) “according to the model, in-depth distances are seen to be about half as large as frontally oriented ones” (once again, the overall finding is of perceptual compression of environmental distance). Similarly, a number of compare or match depth to fronto-parallel width tasks conducted at relatively short distances^8,11,31^ have also produced judgments consistent with perceptual compression of in-depth intervals. For example, in the Loomis, Da Silva, Fujita, and Fukusima^11^ study, in one condition, the observers had to make the in-depth interval physically twice as long as the frontal interval in order for the two stimulus distances to appear equivalent (see Fig. 3a of Loomis et al.). The overall magnitude of perceptual compression in our own study^8^ was comparable to that of Loomis et al.^11^. There have been a number of studies that have previously employed the method of equal appearing intervals or bisection (usually for moderate distances, such as Gilinsky^12^, Norman et al.^13^, and Experiment 1 of Kuroda^21^, but there was one experiment involving long distances beyond 100 m that was emphasized in the current introduction, Experiment 2 of Kuroda^21^). The observers in all of these previous studies also produced judgments consistent with perceptual compression in depth to varying degrees. From this review, there is considerable converging evidence that distances in depth do typically appear perceptually shorter than they exist physically. In reviewing the empirical literature for short and moderate distance ranges, Baird^32^, concluded by saying (p. 280) “These results show very clearly that a far extent must be physically larger than a near extent to be judged equal in size. The exponent of the power function is less than 1.0 showing that this underconstancy is not a simple proportion of target distance. Since this effect is found with Objective instructions, we have here an apparent difference between judgments in a frontal and longitudinal plane”. What the current study and those of Kuroda^21^, Da Silva^22^, and Teghtsoonian and Teghtsoonian^30^ have done is to extend this overall finding to large distances beyond 100 m. What our current experiment has done in particular is to illustrate the large variability that occurs in the long distance judgments of individual observers, and that the power function describing each individual observer’s judgments is quite distinct and unique (see current Figs. 4 and 5).

## Conclusion

Individual observers differ widely in how they perceive long distances in depth beyond 100 m. Some observers make relatively accurate judgments of distances in depth, while the judgments of many others reflect significant perceptual compression, such that physically longer distance intervals located far away are judged to be identical in extent to physically shorter distance intervals located closer to the observer.

## Data Availability

The datasets generated during and/or analyzed during the current study are available from the corresponding author on reasonable request. Results for all individual participants are provided in the figures that accompany this article.
